# Clinical practice during the COVID-19 pandemic: a qualitative study among child and adolescent psychiatrists across the world

**DOI:** 10.1186/s13034-021-00417-y

**Published:** 2021-11-22

**Authors:** Jordan Sibeoni, Emilie Manolios, Emmanuel Costa-Drolon, Jean-Pierre Meunier, Laurence Verneuil, Anne Revah-Levy

**Affiliations:** 1 Service Universitaire de Psychiatrie de l’Adolescent, Argenteuil Hospital Centre, 69 Rue du Lieutenant Colonel Prud’hon, 95107 Argenteuil Cedex, France; 2 ECSTRRA Team, UMR-1153, Inserm, Université de Paris, 75010 Paris, France; 3 APHP, Service de psychiatrie et addictologie de l’adulte et du sujet âgé, Hôpital Européen Georges Pompidou, Paris, France; 4 Service de pédopsychiatrie, Hôpital Robert Balanger, Aulnay, France; 5 IPSE Asssociation, Puteaux, France

**Keywords:** Qualitative methods, COVID-19, Clinical practice, Child psychiatrists

## Abstract

**Background:**

The COVID-19 pandemic has directly impacted the field of child and adolescent psychiatry, affecting all aspects of the lives of children and their families and increasing their risk of distress and mental health issues, especially among children with preexisting psychiatric disorders. Child and adolescent psychiatrists (CAPs) across the world have had to adapt their practice, due to lockdown and social distancing measures. This study aimed to explore how CAPs experienced their clinical practice in these singular conditions.

**Methods:**

This exploratory international qualitative study used the Inductive Process to analyse the Structure of lived Experience (IPSE) approach, which is a five-stage inductive process used to explore the lived experience of participants in depth and to analyze their structure of lived experience. This study took place from March through July 2020 through individual in-depth video interviews. The sample size was determined according to the principles of *theoretical sufficiency.*

**Results:**

39 CAPs from 26 countries participated (age range 32–70 years; 23 women). Data analysis produced a structure of lived experience comprising three central axes of experience: (1) lost in space, lost in time, describing CAPs’ experience of disorganization of their clinical practice in the dimensions of lived time and lived space, (2) the body—of CAPs and patients—underlining their disconcerting experience of both sensory aspects and the non-embodied encounter during clinical practice, and (3) unpleasant emotions, with angst and loneliness the two main feelings coloring their clinical practice experience.

**Conclusions:**

This analysis of the structure of lived experience of CAPs went beyond the sole context of the pandemic and revealed key aspects of what usually organizes CAP clinical practice. It identified two blind spots or conceptual voids within the child and adolescent psychiatry field: first, the intrinsic therapeutic function of a CAP clinical practice and, second, the important diagnostic and therapeutic function of the embodied encounter during CAP consultations. Beyond the context of COVID-19, further research should investigate these aspects to better define what a CAP does in practice and to increase both attractiveness and recruitment in this specialty.

**Supplementary Information:**

The online version contains supplementary material available at 10.1186/s13034-021-00417-y.

## Background

A rapidly growing literature is already addressing the major issues encountered by the field of child and adolescent psychiatry during the coronavirus disease 2019 (COVID-19) pandemic. Several studies have shown a higher risk of distress, domestic violence, child abuse, and mental health issues among families, directly related to the pandemic. They have also shown impacts related to the pandemic concerning anxieties, but also social and economic effects [1, 2]. The issue of child protection during the lockdown was of particular concern in many countries [3, 4] as were the mental health needs and outcomes for children, adolescents [5, 6], and indeed parents [7].

The pandemic has also affected many aspects of the daily lives of children and parents. In many countries, schools closed, out-of-home activities were forbidden or canceled, peer contact reduced to a minimum, and parents at home helped their children (and adolescents) with home or at best remote schooling without the help of any social support system, while working from home themselves. Among adolescents, lockdown led to decreased freedom and privacy. The literature has also acknowledged the greater vulnerability to distress of specific groups: children and adolescents with migrant backgrounds, low socioeconomic status, minority racial or ethnic status, or with special needs and preexisting psychiatric conditions, especially neurodevelopmental disorders [8], including those related to attention deficit and hyperactivity [9], the autism spectrum [10], obsessive–compulsive behavior [11], eating disorders [12], anxiety, depression, substance use, and other types of behavior [13]. While some scholars have reported positive outcomes and opportunities due to this extraordinary situation [14], most studies among children with and without preexisting psychiatric conditions have reported negative mental health impacts [15, 16].

Medical systems across the globe have prioritized prevention and care for the sickest and most vulnerable patients, and the child and adolescent mental health sector has had to adapt by performing medical distancing, defined as a form of social distancing that includes “limiting, whenever possible, patient visits to health care facilities and health care provider contacts with hospitalized patients,” and “also involves patients avoiding or delaying medical care” [17]. Consequently, full closure or reduced services in inpatient, day hospital and outpatient facilities occurred in many places [1]. When inpatient treatment was maintained, major changes occurred that impeded usual care and modified the “therapeutic milieu” [18, 19]. When possible, continuity of care was ensured virtually, with telepsychiatry often implemented successfully or at least rapidly expanded [20, 21]. This remote psychiatric care, however, also raised many issues; these include but are probably not limited to, justice and equity issues, privacy concerns, children’s tolerance for this practice, the risk of being hacked, and the difficulty or impossibility of conducting some therapeutic interventions [1].

And what about child and adolescent psychiatrists (CAPs) themselves? As healthcare providers, sometimes on the front lines, CAPs were also exposed to physical and mental health risks, including but not limited to secondary traumatization, burnout, anxiety, or depression [22, 23]. CAPs have been confronting a major challenge: maintaining psychiatric care of their patients by supporting the increased psychiatric needs of children and adolescents dealing with the psychosocial outcomes of the pandemic. Several child psychiatrists wrote first-person accounts to share with the scientific community what they have experienced during the pandemic—both personal and professional challenges, obstacles, and opportunities [3, 24]. Qualitative methods are also relevant in this context because they can focus on their subjects' lived experiences and views. Until now, only two qualitative studies have been published about CAPs during the pandemic [25, 26]: DiGiovanni et al. [25] investigated the personal and professional impact of the COVID-19 pandemic on the development, practice, and shifting values of 24 CAPs in the USA. Their results emphasized that the pandemic led to “a collective agreement toward the need for implementing change”. Using photo-elicitation, Herrington et al. [26] examined the personal and professional impact of the COVID-19 pandemic on 134 mental health professionals working with children and adolescents in 54 different countries. Their results pointed out important issues about inequalities and social justice challenges, but also showed positive opportunities emerging from this crisis. Both studies explored the global experience of CAPs—not specifically clinical practice—and offered relevant insights for addressing global and local issues among CAPs during and after the pandemic.

Both the lockdown and social distancing measures imposed in many countries to limit the spread of COVID-19 have hindered normal clinical practices. CAPs, all around the world, could no longer maintain their usual framework, as consultations were postponed, canceled, or conducted by telephone or video call. How have CAPs experienced their clinical practice in such singular conditions?

The aim of this qualitative study was therefore to explore the lived experience of clinical practice during this pandemic among CAPs across the globe. Capturing this experiential knowledge was seen not as an end but rather as a means of eliciting concrete proposals to improve CAP clinical practice both during singular and more normal conditions.

## Methods

This exploratory international qualitative study used the inductive process to analyze the structure of lived experience (IPSE) approach [27], a qualitative method specifically developed for conducting clinical medical research that offers concrete proposals (for example, by identifying clinical implications that could improve the quality of care or new research perspectives, or by developing scales that can be used in both clinical and research settings). This approach relies on an inductive process that explores in depth the lived experience of participants and the analysis of the *structure of lived experience*—an expression embedded in the descriptive phenomenological approach and referring to a structure composed by central axes of experience that describe and organize participants’ experiences.

Five stages structure the entire research process: (1) setting up a research group; (2) ensuring the originality of the study; (3) recruitment and sampling, aiming for exemplarity; (4) data collection and access to experience; and (5) data analysis, from the description of the structure of experience to its practical implications.

The report of this study complies with the COREQ guidelines [28]. This study took place from March to July 2020. All participants provided informed consent before inclusion.

### Stage 1: setting up a research group

Our research group included three CAPs (two men, JS and ECD, and one woman, ARL), one woman psychologist (EM), and two medical doctors from other specialties (one woman, LV, and one man, JPM), all experienced in qualitative research methods.

For heuristic purposes (i.e., to facilitate discoveries and novelty), the group’s members were highly diverse, especially in their knowledge, age, and backgrounds. The group worked continuously on reflexivity during open discussions between the researchers, that is, on reflecting on and discussing our roles in the study and its effects on our findings at every step of the research process so that we could each avoid the pitfalls of applying our own preconceptions and assumptions to the material.

### Stage 2: ensuring the originality of the study

Two members of the group (ARL and LV) reviewed the qualitative and quantitative literature systematically and contacted other research teams that were most likely work on similar projects, to confirm the relevance and originality of the study. They verified that no qualitative study had ever been or was currently being conducted on the experience of clinical practice during a pandemic among CAPs. To remain inductive and open to novelty, the other group members had access to this review only after completing the data analysis.

### Stage 3: recruitment and sampling, aiming for exemplarity

The research group defined the inclusion and exclusion criteria (Table 1).

**Table 1:** Inclusion and exclusion criteria

Inclusion criteria	Exclusion criteria
Child & adolescent psychiatrists who have completed their postgraduate training	Trainee or resident
Was in clinical practice during his/her country’s COVID-19 pandemic	Retired doctors
Agreed to participate in the study	No professional CAP activity during the pandemic
Fluent in either French or English	

Participants were selected by both purposive and convenience sampling [29]. Purposive sampling was deliberately intended to attain exemplarity, that is, to select participants who have experienced archetypal examples of the situation under study, and the purposive sampling strategy aimed to include participants who might enrich and add something new to what has previously been found, with maximum variation to enable the selection of participants who differed by sex/gender, age, family status, years of experience, rank in their department, and type of practice. *Convenience sampling sought to facilitate the identification of French- or English-speaking CAPs, through the networks of three psychiatric associations, namely, the European Psychiatric Association (EPA), the European Federation of Psychiatric Trainees (EFPT), and the International Association for Child and Adolescent Psychiatrists and Allied Professions (IACAPAP).*

Sample size was not defined in advance but rather determined by data saturation according to the principle of “theoretical sufficiency” [30]. Inclusion of new participants continued until the analysis of new material no longer yielded new findings; that is, data collection and analysis were complete when the group considered the axes of experience obtained during analysis to be a sufficient explanatory framework for the data collected.

### Stage 4: data collection, access to experience

Two researchers (JS and EM) independently conducted the interviews. They met each participant virtually (by email, or various video meeting software), obtained his/her consent, and collected social/demographic data, thereby facilitating the subsequent research interview, which usually took place immediately after this first contact. They then applied an *open-ended approach* to conduct in-depth interviews. The initiating prompt was “Can you describe in detail a typical working day during the pandemic, from the moment you wake up to the end of the day?” This approach suited our objective of obtaining an in-depth narrative of their lived experience; as the participants recalled their activity, the interviewer frequently prompted them to expand on their feelings, emotions, and thoughts. The participants all accepted open discussion easily; they never focused only on one typical day, but also spoke about other singular and relevant experiences and their general point of view. The interviewers used an interactive conversational style. The interviews lasted from 60 to 90 min. They were recorded and transcribed into anonymized transcripts, which included the participants’ expressive nuances. These transcripts were then analyzed.

### Stage 5: data analysis, from the description of the structure of experience to its practical implications

**Fig. 1:**
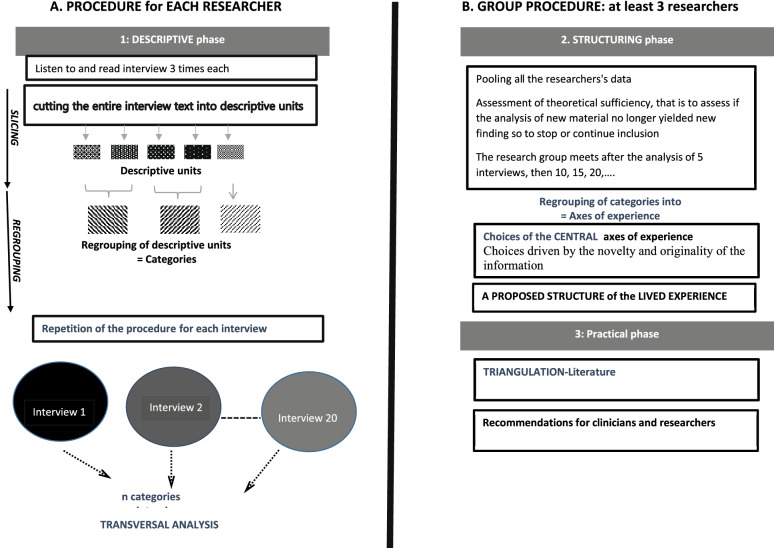
Data analysis procedure. **a** The procedure of each researcher, initially performed individually, corresponds to the descriptive analysis phase, including: (i) listening to and reading the interview several times, and (ii) cutting the text up into descriptive units and then regrouping them into categories. This operation was performed for each of the 39 interviews, which were analyzed transversally. **b** This structuring phase involved a group procedure with regular pooling of the data and analysis, during which the theoretical sufficiency was assessed. During this phase, the axes of experience were produced and the group determined the central axes of experience, which resulted in the proposal of a structure of the lived experience. Finally, the practical phase leads from triangulation with the literature to concrete proposals

The analytic IPSE process is detailed elsewhere [27] and is summarized in Fig. 1.

It relies on an inductive, phenomenological method in two stages: one of independent work by three individual researchers, and one of pooling the data by the group. The individual procedure consisted of three qualitative researchers (JS, EM, and ECD) independently and simultaneously conducting a systematic descriptive analysis aimed at conveying each participant’s experience. For each interview, this involved: (1) listening to and reading the recorded interview and transcript several times; (2) exploring the experience word by word, that is, cutting the entire text up into descriptive units; and (3) regrouping the descriptive units into categories. These stages were conducted with the help of QSR NVivo 12 software.

During the group process, all group members—also familiar with the data after listening to and reading all the interviews several times—met after every five interviews to conduct the structuring and practical phases of analysis. In the structuring phase, the categories are regrouped into axes of experience, constructed such that each can be linked to its subjacent categories and the structure of lived experience characterized by the central axes thus determined. The practical phase is a process of triangulation with the data in the literature to identify the original aspects of the results and draw practical implications from them.

### Criteria for rigor in the analyses and patient and public involvement

We used several criteria to ensure the rigor of the analysis and the trustworthiness of the results: triangulation, attention to negative cases, reflexivity within the group process, and feedback from “subjects of the experience” by presenting the research to a group of CAPs (N = 20) who had been approached but not included in the sample. In practice, we asked them to react to the results in four separate group video meetings, in which all recognized their own experience in the structure we proposed. Their reactions included agreement as well as surprise that some findings put aspects of their lived experience into words for the first time.

## Results

**Fig. 2:**
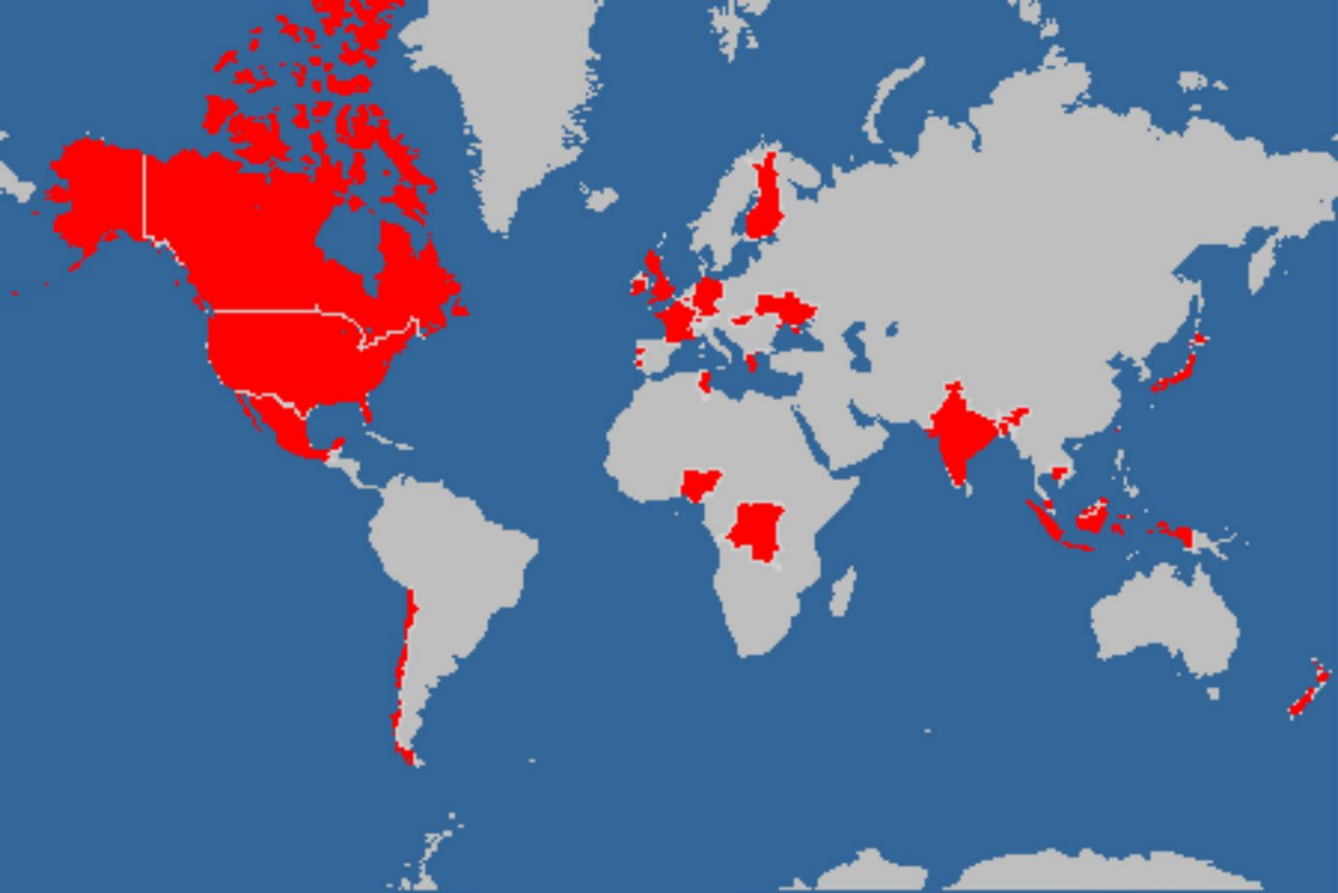
Countries included

We included 39 CAPs from 26 countries, including 23 women; respondents ranged in age from 32 to 70 years (Fig. 2). Participants’ general characteristics are presented in Table 2, and detailed characteristics for each participant are available as Additional file 1: Table S1.

**Table 2:** Summary of participants’ characteristics

Characteristics	n (%)
Gender	
Men	16 (41)
Women	23 (59)
Mean age, years	45
Clinical position	
Head of department/medical director	6 (15)
Senior position (fellow, physician)	17 (44)
Chief resident	3 (8)
Staff consultant/visiting staff	8 (20)
Consultant	5 (13)
University/teaching position	12 (30)
Professor	2
Assistant professor	3
Associate professor	2
Trainee director	1
Lecturer	4
None	27 (70)
Clinical practice	
Outpatient	39 (100)
Inpatient	11 (28)
Day hospital	3 (8)
Liaison-consultation	4 (10)
Research activities	16 (41)
Private practice (full or partial)	11 (28)
Psychotherapy/psychotherapy training	8(20)/6(15)

Data analysis produced a structure of lived experience formed by three central axes of the experience: (1) lost in space, lost in time, (2) the body—of CAPs and patients, and (3) unpleasant emotions. Table 3 presents relevant quotations (from the interview transcripts, some translated from French into English for the sole purpose of this article).

**Table 3:** Quotations

1. Lost in space, lost in time	
1.1 Lived space	Q1, P2: The first thing I did when I learned about lockdown was to go back to the hospital to back up to my files… they said, “from tomorrow, you should not come, because the hospital is closed” Q2, P10: I only work from home and it’s very difficult. My husband is also working from home and now, we don't have the person who usually comes to take care of our baby Q3, P28: (every day, it’s going to be where you are right now, on this chair, on this computer?) Yeah, on this chair, on this computer, on this butt Q4, P5: They told us, “from now on you are hospitalizing patients at their home”… patients stay at home and we contact them remotely every day Q5, P32: We would still have our 8:30 in the morning meeting, but we had to split into two different groups… And we had, on the different chairs, we had a marking of where you could sit on the floor Q6, P14: I also have to go to the COVID area, because in our hospital we divided the department space to a COVID area and a non-COVID area. So, the child psychiatry [department] also had to provide surface to the COVID area… We have to wear the full level-three PPE. It's tiring, very tiring because it's very hot… At the beginning, I was a bit reluctant to do that
1.2 Lived time	Q7, P3: Before, my schedule was well defined and I have to admit that currently my days are more chaotic… I mix private and public practice on the same day and sometimes I get completely lost Q8, P20: Many times the parents (of teenagers followed in psychotherapy) want to see you, and then you have to inform the parents, and you lose time Q9, P14: Now in Corona conditions, our government and our hospital limit the working time… So I work in alternate days. If I work today, then tomorrow I get a holiday, then the next day I have to go to the hospital Q10, P34: We don’t know how long we will have to continue like this… one year, two years, nobody can tell. So we are here, working like this and waiting, and waiting… It’s a never-ending story Q11, P2: We were just told that the hospital is going to close, and that they would let us know how to take things further. Only emergency services would be available,… everything was canceled… We had a long waiting list, so we had appointments scheduled until June or July, and we had to cancel everything from March, until now and maybe after, who knows?
2. The body—of CAPs and patients body	
2.1 Sensory aspects Touch	Q12, P28: There's some cases where I find it really difficult and particularly, again, when you're dealing with kids with neurodevelopmental disorders where I can't do physical exams…I like listening to somebody’s heart, I like feeling their pulse, I like doing a neurologic examination because I often find things that other people haven't found. I'll see patients referred to me by neurologists or pediatricians or others, and I find things that they didn't find. It's much harder to do that… there's fun in doing that too, by the way. But I can't Q13, P21: We could not do the physical work anymore, all the holding, all the care, the baby’s sight, seeing them together, breastfeeding, we need to support this, it's very important during the first weeks, I don't necessarily touch them directly but all of this from a distance is way more difficult, because during a teleconsultation or through whatsapp, you see them in a small square. It’s more delicate to give proper advice and to assess the situation Q14, P8: I fear that we somehow we're going to continue being afraid of contact with people… that's going to be an issue for me if everyone wants to keep a lot of distance and space in between Q15, P2: Because, generally in a child psychiatry clinic, we generally give a handshake, or we hug the child, or you pat the child on the back, and should we stop doing all that? We had all these kinds of practical discussions of how we're going to manage Q16, P9: I mean you're trying to keep it always as you can, but you know with the kids they just come and crawl on your lap and what can you do? You just go with the flow
Sight	Q17, P32: I was in the mask and the visor; that was particularly weird because I can see their face and they can't see me. So that felt deeply unsatisfying to me and il felt like a very, to be blunt, not the robust comprehensive assessment I would like, in terms of what I can give to my patient Q18, P01: With the distance, there was something emotional that could not go through. Even a patient told me “but if we speak on the phone, you won't see my emotions.” And even through video, I could see the face, but not perceive the emotions Q19, P20: Sometimes, the clue is in the face you know, the way they look when they tell you a joke or make an ironic comment. But 2 m plus the mask, you miss it, you don't spot it… and you end up looking like a fool ‘cause you did not get it was a joke Q20, P06: my gut feeling was, we do our work with some automatism, the analysis of the information, the semiology, the nonverbal communication… And you become aware of all of this only when precisely you can't do it, you can't have access to that Q21, P28: I can't look for dysmorphology and just sometimes putting your hands on somebody tell you something very different about tone and spasticity and things like that, that you just can't see. You can see a head and shoulder tic, but you may not be able to see their hands or their legs or… and even then, you might see only one part of it, or they… so, some of those things it's much, much more difficult to assess on Zoom or whatever platform you use. Well, it's incomplete and uncomfortable because we can't do it… Q22, P30: I prefer using the phone with my patients. At least they don't expect me to read their facial expression to understand what they really mean. Sometimes the image can freeze or, because of a bad connection, there is a gap between image and sound… It's not reliable enough Q23, P06: It was not possible through video, because body image was precisely the clinical issue with my anorectic patients, I have two and doing video consultations was problematic so we did it over the phone
Hearing	Q24, P15: With some adolescent patients who did not want to show their faces on the camera, it was difficult, especially with one of them who rejected categorically telepsychiatry principles. At the end, I could only speak with his mother and did not even hear the sound of his voice Q25, P01: Some teens refused to speak to us, we knew why… Because their parents were standing next to them. There's not the separation we used to have…
2.2 A non-embodied encounter	Q26, P4: Of course, there are some things that you need to do in person… Surely there are some things that we can't do, like a physical exam or… But 98% of what we do you can do like this. Is it different? Yes, it's different but we need to adapt Q27, P20: I don't’ like it, I feel I can't really contain their anguish by just appearing on their phone Q28, P3: I had a case last week and they, the parents and the patient, didn't do anything that I recommended. I guess I'm less convincing on ZOOM than on reality Q29, P3: Now I have a safety net now, I always ask for a parent to be around when I do a consultation via ZOOM Q30, P9: It's meeting via screen; it's lacking the essence of the meeting… face to face… all the bodily work that we do when we meet our patients or with the family, containing the emotions or feeling the energy, the atmosphere… and assessing or finding things between the words or outside the words. So, that of course, is missing Q31, P24: When someone is someone is psychotic, you feel it, you smell it when he gets into the room. You feel it in your body, like, “Ah, something's not okay.” And it's kind of hard long-distance. And what I haven't done, but I think it would be really hard, but I haven't, is autistic disorders. I haven't had that in this quarantine, but that might be difficult I'm thinking Q32, P34: all of our training programs have transitioned to remote learning, remote teaching completely. We don't have any in person outpatient care in child psychiatry… I think some trainees are a little concerned about the quality of their education, how they're going to be impacted if they're not going to see these kids
3. CAPs’ emotions	
	Q33, P17: I saw an opportunity for the families to find resources. I was quite calm about it and, with the exception of one family, all the others did pretty well. Family competency is confirmed! Q34, P25: Because of the family crisis causing effects on the patients, if there’ a family crisis, not only have they been through depression problems with some of the family but it is also being exacerbated by economic issues, which are not federal in Mexico in these days. Jobs have been lost. Most of them are middle class employees and they have therefore affected many of our patients now. It's very sad Q35, P01: and also the effects of this peculiar period, we thought we would see a lot of patients with OCD and anxiety. A lot of teenagers, because teenagers would be stuck at home with their parents. Not an obvious situation, we all know that. That was our fear
3.1 Angst	Q36, P05: I was highly anxious myself at this moment… the first 15 days, it was really general anxiety. I was like: what is happening? Are we in danger? Those first 15 days it was more handling my own anxiety Q37, P22: They were, but I had a pool of patients, let's say there were about two handfuls of patients, these were the patients that I was worried about because these were depressed patients… there’s also the adolescent patients that I was worried about. That day some of them had ADHD, some of them ADHD and depression, so I was worried about how they were going to cope Q38, P5: This kid did not have access to technical means to…, not everyone has internet you know, or a computer at home… and it was very difficult to reach her on the phone. We tried but no one was answering. When it is discontinuous like that, there is less presence, It does not make sense for them I think. And physical presence, I mean when you are not well, it is not about what you say or do, but it is about being here. And this is a big part, even the main part, of our job: be there, at the end doesn't matter what you say as long as you are here for them, even in silence. There is this need for physical presence, to really see them Q39, P1: For instance, kids with anxiety, with school refusal issues, what we used to call school phobia… the fact that they are doing well during this crisis, I think we need to think more about it Q40, P9: More self-disclosure because some parents and even some kids allow themselves to ask about how I am doing, for example, when ending the session, they could say that they hope my family stays healthy. Or be safe. Or “be safe you and your family”, so stuff like that that they wouldn't normally do Q41, P20: Honestly, I don't know if you I call it “therapeutic consultation” when all what we can do is to chat on the phone and send the prescription by mail. I mean, of course it must have had a therapeutic effect, but definitely, it was not the same… I wouldn't say that I continue to “treat” them during the lockdown, what I did was keeping in touch… at the end of the day, it’s not much Q42, P3: No, I didn’t make them pay… I would not feel comfortable with this
3.2 Loneliness	Q43, P6: It was empty, a phantom-like ambiance… we felt very isolated since all the meetings were postponed, canceled, or transformed into ZOOM meetings. All the training part was canceled
Working alone, without colleagues	Q44, P16: It was the recommendation that people not stay at home so that all the staff of the polyclinics came to the polyclinic, but then we had to operate with the remote connections. So we really tried not to meet in the kitchen or in the cafeteria or in the relaxing room, but instead of that, we had coffee at our desk and all the meetings between the staff with remote connections via Zoom or Skype… Kind of only coming here to work and not to interact Q45, P34: I probably leave the house around 7:30, I get here around 8:00 AM… and I just have Zoom after Zoom. I have Zoom fatigue. I’m constantly like boom, boom, boom. Usually one meeting kind of bleeds into another… A full Zoom day Q46, P1: We work from a distance, and way less than usual. We went from full days of work to keeping few hours per days. And at home, it is more difficult to work. I remember when we did the first meeting online, I said I want everybody with the camera on, I don't want anyone in pajama, not shaved… I have this team responsibility and it was very important that the team feels the solidarity and the bond between us, not everyone in his little corner Q47, P15: I woke up, I was going to work for practical reasons, my partner was working from home and was always on the phone. It was not possible Q48, P18: I had a full agenda with a lot of meetings and contacts with colleagues between the meetings Q49, P28: I think the hard part is that there's no separation between work and the rest of your life… it's tiresome. You don't get up and walk around as much. There's not as much writing in your day and you miss direct contact with your colleagues. Although, we have meetings. We've written three grants in this time. You meet with your grant-writing team and all of that. In fact, I'm working on my fourth grant now. So, a grant a month, which is pretty good. But it's tiresome but it feels good to interact and work together on these projects Q50, P01: One interesting point, we could invite people from the all world to our meetings, as they were online Q51, P6: Some disappeared from our radars. Child protection services, they were only teleworking and only by phone. It was not organized at all and they completely stop the interventions. Some situations, without the support and intervention in the presence from child protection services, families don't show up Q52, P33: As I was saying, the day hospital is closed and we don't know yet when it will reopen… so the day hospital patients will be followed in the outpatient clinic, and for the ones that colleagues want to address for day-hospital, it is even impossible now to even put them on a waiting list
Working alone without the other childcare professionals	Q53, P6: I have one particular situation that worried me a lot. I had to hospitalize a patient… I had the feeling I was all alone, dealing with this situation, I tried to call everybody and no one was available, I mean no one was even answering the phones. Contacts were through mailing only except in some urgent situation it is not possible to do it through e-mails Q54, P27: We were concerned that the children were likely going to regress because they're supposed to continue or start therapy and all, but because we were not able to access, we were afraid that we are likely going to lose some milestones by the time they could benefit for specific care Q55, P32: I can see them, but they can't see me. So one of the things that I suggested to our nurses that need to make some badges, a big picture of what we look like without the PPE, to help our patients see us, see our face Q56, P8: Those kids, I'm still seeing them in person. I take them for walks… They are not allowed in the program. So I meet them outside the program in the street Q57, P6: With one patient for instance, we played chess together. We both had a chessboard and we played like that. Then he suggested we directly play online and it was easier
Resilence and creativity	Q58, P2: In fact, we actually thought families would have difficulty managing the change, taking care of them throughout the day because day care [centers] are closed, therapies are closed. They don't have… I mean, I predominantly see autism and developmental disorders in the hospital, so I actually expected a lot of things to go badly. But, we had many parent interviews, we had Webinars with parents, we had online meetings with parents, groups, asking how they're all doing. Surprisingly, most of them seem to be doing really well, probably because both parents are at home, there is a lot of family support, and now they're actually working almost as therapists, they're actually doing home-based intervention programs, they are the only therapists available Q59, P24: They're at home, so the mom said, “Why is she always goes to the bathroom after we eat or stuff like that?” The quarantine makes the parents more responsible and that they can spot more of the symptoms. That's clearly happening with eating disorders. They are getting aware, or they cannot minimize that a girl is jumping three hours at home. You can see it, it's not like sometimes they take them to the gym and say, “Okay, she stood at the gym, maybe she was with her friends.” And now no, now they are looking at them Q60, P9: It is going very well with the parents… We are completely on the same team, working together… Paradoxically, the pandemic is doing well for the alliance with the parents Q61, P12: I mean I always ask my patients what they prefer. If they want to have the session with their parents around, or if they want to talk to me alone. And I find that even a lot of the parents wants their teens to talk to you alone, and usually the kids end up going to their bedrooms with the phone or what-ever, or they go to the basement, go to kind of a quiet space for them. Yeah, but some still want to be around their parents

### Lost in space, lost in time

The participants considered that the pandemic, lockdown, and social distancing restrictions deprived them of their usual framework, producing a singular and confused experience of their clinical practice that affected both their lived space and lived time.

#### Lived space

CAPs reported radical changes in their workplace space. Some explained that their hospital or their department had closed: they had to work from home, far from their office, and without access to either their patients’ files or spaces for formal or informal exchanges with colleagues (Q1). Many insisted on the difficulty of working in an environment not intended for that purpose, such as “in the kitchen” or “in my kids’ bedroom”. They underlined the lack of boundaries between their professional and their personal/family spaces, especially when the rest of their family was also at home (Q2).

Other participants continued to work on site, yet they all experienced the workplace environment and space as completely different, stating that “the hospital was not the same, from the parking lot to our shared office” or that “everything looks different”—: outpatient and home hospitalizations were only done in teleconsultations by telephone or video (Q3, Q4), staff meetings took place in very large rooms or were split into two groups (Q5).

The workplace space was redesigned either to meet safety requirements or to become a COVID unit (Q6). In the latter case, participants described their apprehension and reluctance to stay and work in such a unit, pointing out “locked doors that I've never noticed before” or how “everyone looked as if they’re in an operating room.”

#### Lived time

Similarly, most of the participants experienced time differently during this period. This was especially true for the CAPs with mixed practices—part hospital-based and part private, or in several different places. Before the pandemic, their time schedule was well organized, with days or half-days dedicated to one type of practice or one place. They then experienced an “anarchic” or “chaotic” time schedule, with no time markers helping them to compartmentalize their different practices (Q7). Other participants reported a feeling of wasting time, while they were talking (Q8) or obtaining information about the pandemic rather than practicing child psychiatry. One mentioned that she was, for example, “easily spending hours every morning reading public health and epidemiological reports and then I’m late for my ZOOM meetings”. Others felt that temporality was imposed on them in their daily professional life (Q9), reporting that the “hospital administration” or their “department head” ordered them “when to come and work,” “when to stay and when to leave,” “when to work from home” or even “when to be with my kids". They considered this imposed schedule as a supplementary and unnecessary obstacle: “It’s already quite difficult and tiring to work like this without them *putting a spoke in the wheel* telling you when to come to work”*.*

Overall, this peculiar period was experienced as a time of “uncertainty” and “unpredictability” with an “unknown ending” (Q10). Participants said they were entangled or trapped in a “here and now” moment and found themselves with problems in their practice, for example, for rescheduling a face-to-face consultation appointment, as they had no idea when in-person visits would be allowed again; or planning a hospitalization, given that many inpatient units had been closed or converted into COVID units; or meeting with partners, as most of the other childcare professionals were only working remotely Q11).

### The body—of CAPs and patients

All participants emphasized atypical, disconcerting experiences related to their own body and those of their patients. These concerned sensory aspects as well as the non-embodied encounter.

#### Sensory aspects

##### Touch

Most participants found themselves practicing child psychiatry without being able to touch their patients, having “zero contact”. Some, in their daily practice before the pandemic, used to perform a physical examination to complete their assessment. They felt frustrated that they could no longer do so and considered that their assessment was therefore incomplete (Q12, Q13).

Other participants described the importance of physical contact in the patient-physician relationship, particularly with children, during the consultation. It was difficult for them to endure this absence of physical contact (Q14). They could not, for example, even shake hands with patients and families—“once, I started the process of hand shaking and it was the adolescent who stopped me, ‘no doctor, we can’t,’ he said”. Similarly, they could not hug younger children, or stand next to them and put a hand on their shoulder to reassure and comfort patients in distress (Q15). A few even said they could not restrain themselves and did not avoid physical contact with patients, especially with young children (Q16) and distressed patients—“she was crying, what was I supposed to do? Not give her a tissue?”.

##### Sight

Three types of clinical practice were possible: not seeing the patients at all (consultations via telephone), not being able to perceive their faces and facial expressions (in person but masked), or seeing only the face/the upper body (telepsychiatry).

Thus, in some countries, especially low- and middle-income ones, participants continued to receive patients and families in person, while respecting regulatory distances and with everyone wearing masks (except for children under six). Not being able to perceive the entire lower part of the face made it difficult for CAPs to assess and interact with patients (Q17). They could not grasp the emotional dimension (Q18). Without seeing the patient’s face, they sometimes did not understand the irony or cynicism of a remark by a teenaged patient (Q19); some then realized how much they intuitively relied on facial expressions and nonverbal communications for their assessments (Q20).

On the other hand, participants working with telepsychiatry mentioned the opposite problem—seeing only the face. They could not observe the rest of their patient’s body and spot psychomotor signs and symptoms and thus had a global feeling that the assessment was incomplete (Q21). Some participants even preferred telephone to video consultations, because the latter device gave them the illusion of seeing and reading emotions and nonverbal expressions correctly, although incomplete views, poor image quality, and bad sound-image synchronization meant that this was not always true (Q22). Some CAPs chose to continue their follow-up only by telephone with patients they already knew, especially when their condition involved body image: “basically, I just did what I used to do from time to time before, making phone calls to my patients” (Q23).

##### Hearing

Telepsychiatry appeared particularly limited with younger children and children with certain conditions, particularly neurodevelopmental disorders. Participants could not speak directly with and listen to their patients but often talked only with the parents (Q24)—“working only from what the parents can tell you about their kid”. Similarly, they noted that some adolescents could not speak freely because of the lack of privacy and the risk that their parents could listen to everything they had to say to the psychiatrist (Q25), preventing the CAPs from hearing everything their patient would have had to say to them in a normal appointment.

#### A non-embodied encounter

While some participants were very satisfied with telepsychiatry and its practical benefits—“saving time”, “being on time”, “avoiding a commute”—all without disrupting their practice (Q26), many felt powerless to help, act, or make therapeutic decision without being in the same room as their patient (Q27, Q28). Several participants mentioned their fear that adolescents could self-mutilate during a telepsychiatry session and that they could not do anything about it. Others had experienced such events and adopted a systematic approach of arranging for an adult to be present with their patient at the time of the video consultation, which of course made privacy impossible. Some also reported a sense of helplessness when a patient told them they had stopped taking prescribed medication (Q29).

Without an embodied encounter, many CAPs felt that they could not “grasp the ambience” or “atmosphere” of the consultation; in other words, they could not “read the room if we're not in the same room!” (Q30). Here, again, this non-normal situation made them aware of how, in their usual practice, they intuitively relied on their perception of the ambience to assess the patient, formulate a diagnostic hypothesis, decide on course of action, and build a therapeutic alliance (Q31). Finally, several participants also commented that this nonembodied consultation directly impaired the training of medical students or CAP interns to conduct interviews and develop doctor-patient relationships. Students for example could not be physically present at consultations: “only on ZOOM and I don’t think they could grasp that much of what was happening”. Similarly, interns conducting the consultation themselves could not receive direct supervision from senior CAPs (Q32).

### Unpleasant emotions

Participants shared many of the emotions and feelings they were going through during this particular period. Some respondents showed some excitement about opportunities generated by this occasion: families discovering their own resources (Q33), effective and creative “research collaborations”, “waves of solidarity”, among colleagues and between professionals and their patients.

The discourse of many others, however, was imbued with sadness about the pandemic’s health, social, and economic consequences (Q34). Most of them worried about significant adverse outcomes on the mental health of individuals in general, children and adolescents in particular (Q35). Two feelings/emotions prevailed: angst and loneliness.

#### Angst

All the participants recognized that this situation induced anxiety in everyone, including themselves. Many mentioned being “anguished”, “anxious” or “worried” for themselves, their loved ones, and their patients (Q36).

They were concerned with their patients' psychiatric state, especially in countries under strict lockdown where schools and outdoor spaces were closed. They were particularly concerned about families in constant conflict with dysfunctional patterns (Q37) and about patients they could not reach, imagining the major difficulties the latter might have to deal with in their daily lives (Q38). When they finally reached them, they were always surprised about the relative stability and sometimes even symptomatic improvement that patients and families described (Q39).

They were fully aware that reaching out and interacting with their patients, over the phone or through video, was mostly to “reassure them and reassure myself”, “check in”, “stay connected”, “continue to bond”, “maintain their bond”, or simply “keep in touch”. This mirrored the concerns from patients and families toward them. On that topic, many participants mentioned they were more forthcoming with self-disclosure than usual and did not hesitate to talk about personal aspects of their lives with their patients (Q40).

Finally, many CAPs interviewed even wondered about the therapeutic dimension of their work during this crisis (Q41). Some even refused to charge for their private practice consultations, considering that it was not, strictly speaking, a consultation (Q42).

#### Loneliness

##### Working alone, without colleagues

Many participants found it difficult to work alone (at home or in their office), without the presence of colleagues around them, that is, without meeting them in formal or informal contacts (Q43). They became aware of the importance of “small talk” and “coffee breaks” between colleagues during the working day as sources of renewal and restful moments, but also of the sense of security conferred by the mere fact that colleagues were seeing patients in the adjoining offices (Q44). Many participants felt exhausted working alone on their computers all day (Q45).

All participants, especially those working from home, endeavored to maintain regular contact with their coworkers (Q46). Many preferred coming to work at the hospital to conduct their video and telephone consultations, to keep their professional and personal lives separate and to interact with colleagues (Q47). Others multiplied meetings online with colleagues and spent several hours per day communicating by phone or email with other professionals (Q48). Some explicitly stated that these actions purposely sought to ease their feeling of loneliness and “creat[e] the illusion of social life through work” (Q49, Q50); other realized during the research interview that “maybe I don’t really need to spend that much time on the phone with my colleagues… maybe I use it as an excuse, you know, because I miss them, I miss human contact”.

##### Working alone, without other childcare professionals

Participants also mentioned the lack of relationships with other childcare professionals (pediatricians, school nurses, primary care professionals, child protection workers, etc.) (Q51). They reported that it became impossible to refer patients for full-time treatment such as a day hospital or inpatient care or to specific therapeutic interventions (such as group therapy, psychomotor therapy, or speech therapy) (Q52). Most of the time, a multidisciplinary approach was difficult if not impossible, and participants felt isolated in their clinical practice. Many participants were concerned about delayed diagnosis and regression/stagnation in the absence of specific treatment, especially for patients with ASD (Q54). Finally, many had to work without the support of child protective services, even though situations would, in normal times, have required it. They were often the only childcare professional available for such situations and found this isolation very hard (Q53).

##### Working alone: resilience and creativity

Participants explained how they used creativity to adapt their practice to continue doing their work alone (Q55). Many found “outside the box” and innovative strategies such as meeting patients outside of the hospital to take a walk with them (Q56) or playing on-line games with a patient during a video consultation (Q57).

Instead of feeling alone, many began to work with parents. They now regarded the latter as the primary source of information about their child’s condition and daily life and as the only “therapist available” to treat him/her (Q58). Many said that they were working “intelligently” with parents, giving them specific tasks and exercises to do with their child, and systematically considering their perception and opinion of the child’s difficulties and suffering (Q59).

As a result, they noticed improvement in the therapeutic alliance and relationships between parents and themselves and considered that this “collaborative work” had direct positive effects on the child’s clinical state (Q60). Some participants reported that this situation had created a perfect opportunity to discuss further important issues such as autonomy and intimacy with both the adolescents and their parents (Q61).

## Discussion

This qualitative study aimed to capture the lived experience of CAPs’ clinical practice during the COVID-19 pandemic. The structure of their lived experience is characterized by spatial and temporal disorganization, nonembodied encounters with unusual sensory features, and the unpleasant emotions they endured, mainly angst and loneliness. Some of these aspects have already been described in the literature among workers during the COVID-19 pandemic in general, and healthcare professionals in particular [23, 31].

Our results present these CAPs’ perceptions of how their clinical practice was disorganized by the pandemic and the working conditions that accompanied it, especially the loss or impediments of many of its normal or usual features: lived-time, lived-space, colleagues, sensory features, embodied encounters, and connections with patients. These contrasts give us access to what usually organizes their clinical practice and structures their encounters with their patients and the care they provide.

Beyond the cultural differences and organizations of health-care systems, the practice of child and adolescent psychiatry is universally based on one or more meetings between the child, his or her parents, and the psychiatrist. During the meeting(s), the CAP observes, questions, and interacts with the child and family. A consensus defines the purpose of these meetings: to glean elements of the history of the child and family, to assess the child’s signs and symptoms, as well as family and social interactions, and thus to be able to diagnose and treat “disorders of thinking, feeling and/or behavior affecting children, adolescents, and their families by providing comprehensive care.” The definition of CAP work by the UK National Health Service emphasizes two aspects: the multidisciplinary and multiagency approach, and the fact that child & adolescent psychiatry “combines the rigours and science of medicine, with the art and creativity of therapy” [32]. However, our results suggest that during the first months of the pandemic CAPs experienced the disorganization of their clinical practice.

First, the main concern of all the CAPs interviewed in this study was to make sure that they did not to lose their connection—virtual or real—with patients and families, although only some considered this work of “staying connected” to be part of the treatment, while others did not. Second, the pandemic made the multidisciplinary and multiagency approach next to impossible. CAPs had to work alone, without colleagues, without specific interventions for their patients, and without the collaboration of other childcare professionals. Third, CAPs could not work with either their *lived body* (to feel, to communicate, to convey a feeling of security, to act) or with the body of their patients (to reassure, to feel, to intuitively react) in their clinical practice. Our results suggest that the absence of an embodied encounter between the CAP, the patient and his/her family, plus the partial loss of some sensory functions, contributed to the disorganization of CAPs’ clinical practice.

Metaphorically speaking, we could say that CAPs experienced what it is to be *a conductor without an orchestra, but also without a live audience, and without the ability to perceive all the aspects of the music*. The participants all wondered if their clinical practice during these first months of the pandemic still had either a therapeutic function—that is produced an effect directly related to the treatment goal or that directly alleviated patients’ distress or symptoms. While some adapted with creativity to their abnormal situation to maintain this effect, others were highly skeptical about the therapeutic function of their clinical work during the pandemic. This is an original result of our study, since the literature is devoid of data on the topic of this therapeutic function of CAP clinical practice, in general and certainly during worldwide or even local pandemics. There is in fact some confusion between the roles of psychiatrist and psychotherapist, since CAPs might have received psychotherapeutic training not to become psychotherapists but to be able to use psychotherapeutic techniques in their clinical practice [33]. An abundant literature has shown that a non-negligible part of the variance in the outcomes of psychotherapy of children and adolescents might be explained by the person/personality of the psychotherapist [34, 35]. Yet, in our sample, only 20% of the participants were also psychotherapists and 15% more had received formal training in psychotherapy. What about the other 65% then? This intrinsic therapeutic function of a CAP clinical practice appears to be a blind spot or a conceptual void in the field. In our results, however, the loss of this therapeutic function due to the disorganization of their clinical practice appeared to be a concern shared by all the participants.

Similarly, the embodied encounter in this field might also be considered a blind spot in clinical practice. Recent literature on the success of telepsychiatry implementation in the field could produce the inaccurate impression that CAP clinical practice does not necessarily require the bodies of both CAPs and patients, except for conducting a physical examination. Our results suggest otherwise: CAPs working for the first time without the possibility of an embodied encounter, due to the pandemic, realized how the latter structures their practice for both diagnostic and therapeutic purposes during consultations.

Further research should investigate these two blind spots of CAP clinical practice to better understand how the encounter with a CAP can be intrinsically therapeutic. The concept of *transformative experience*, as defined by Paul [36], could be used in such research to explore how this encounter can be an *epistemically transformative* experience, providing knowledge and understanding previously unavailable and inaccessible [37], but also *personally transformative*, fundamentally changing both patients’ and parents’ values, preferences, desires, and identities.

CAPs are in short supply today, with a lack of attractiveness and downward trend in recruitment in many countries [38]. A recent study showed that the two main reasons given by trainees for choosing to specialize in this field were “working with children” and “interest in psychotherapy” [39] Highlighting the direct transformative and therapeutic functions of the clinical practice of child psychiatry and thus modifying the definition of CAP work could give a clearer picture of the daily work of CAPs and potentially increase the attractiveness of and recruitment in this specialty.

### Study limitations

First, this study focused on the common experience of the sample and did not try to encompass the influence of social and economic aspects and their differences between participants. This would have required a quantitative study comparing subsamples of CAPs from several countries in the time of COVID. Similarly, our results produced a common and decontextualized structure of experience. Exploration of the same research question in each participant's professional context—which depends mostly on the organization of the country’s medical and mental health systems and its economy—would produce different results.

Second, our results were common to all the doctors, regardless of the severity of the pandemic and the strictness of the social restrictions in their country. Further studies should explore in depth the lived experience of CAPs in the countries most exposed to COVID-19 and where social restrictions have been most stringent.

Third, this study focused on the sole perspectives of CAPs. Further research should explore the lived experience of patients and their families to cross the perspectives and better understand the dynamics of patient-physician relationships during the pandemic.

Finally, our results do not include any cultural features. Since this study had a cross-cultural setting, we would have expected to collect some culturally specific data regarding the lived experience of the participants. One possible explanation for this absence of cultural aspects in the participants’ narratives might be the language of the interview itself, most of the time not the participants' (interviewers and interviewees) mother tongue/native language. Further qualitative studies focusing on one cultural context would be necessary to better assess and understand the role of culture in CAP clinical practice.

## Conclusions

In this qualitative study, the COVID-19 pandemic’s impact on CAPs' work enabled us to uncover and make explicit two essential aspects of their clinical practice, that is, its intrinsic therapeutic function and the embodied encounter. Both could potentially justify considering the encounter with a CAP as a transformative experience for patients and their families.

## Supplementary Information

**Additional file 1: Table S1.** Characteristics of each participant.

## Data Availability

The datasets analyzed during the current study are available from the corresponding author on reasonable request.

## Abbreviations

CAP: Child & adolescent psychiatrist
COREQ: COnsolidated criteria for REporting Qualitative research
COVID-19: Coronavirus disease 2019
IPSE: Inductive process to analyze the structure of lived experience
