# Supplementary material for: Clinical practice during the COVID-19 pandemic: a qualitative study among child and adolescent psychiatrists across the world
Source: Child Adolesc Psychiatry Ment Health. 2021 Nov 22;15:68. doi: 10.1186/s13034-021-00417-y (PMC8608359; doi:10.1186/s13034-021-00417-y)
Supplement: Supplementary file 1 — Additional file 1: Table S1. Characteristics of each participant. [file 13034_2021_417_MOESM1_ESM.docx]

| **Table S1. Characteristics of each participant** | | | | | | | |
| --- | --- | --- | --- | --- | --- | --- | --- |
| N | age | gender | Country | COVID-19 country situation  At the time of the interview | Year of CAP graduation | position | activities |
| 1 | 48 | M | Tunisia | lockdown | 2004 | Head of department | Public outpatient and inpatient  Private practice/psychotherapy  Research |
| 2 | 33 | F | India | Lockdown | 2017 | Senior clinical researcher associate | Outpatient (public and private practice)  Research  Lecture |
| 3 | 51 | F | Ireland | Lockdown | 2012 | Consultant | Outpatient (Public and private practice) |
| 4 | 54 | M | USA | Lockdown | 1995 | Professor  Medical director of inpatient unit | Inpatient and outpatient  Research |
| 5 | 42 | F | Belgium | Progressive ending of the lockdown | 2011 | Chief of resident | Inpatient and outpatient  psychotherapy |
| 6 | 32 | M | Switzerland | Progressive ending of the lockdown | 2018 | Senior physician | Outpatient  psychotherapy |
| 7 | 40 | F | Taiwan | No lockdown  Social distancing | 2013 | CAP visiting Staff | Inpatient and outpatient  Research |
| 8 | 45 | F | Canada | No lockdown  Social distancing | 2008 | Associate professor | outpatient  Residential care |
| 9 | 39 | F | Finland | Lockdown | 2015 | Senior fellow | outpatient (public and private practice) |
| 10 | 36 | F | Chile | Lockdown in Santiago | 2013 | Senior physician | Liaison-consultation  Private practice  Lecture |
| 11 | 39 | M | Bangladesh | Lockdown | 2017 | Assistant professor | Inpatient and outpatient |
| 12 | 46 | F | Canada | No lockdown  Social distancing | 2012 | Staff consultant | Inpatient and outpatient  Residential care |
| 13 | 44 | F | Taiwan | No lockdown  Social distancing | 2010 | Assistant professor | Outpatient |
| 14 | 42 | F | Indonesia | Lockdown-like  Social restriction | 2014 | assistant professor | Inpatient and outpatient |
| 15 | 33 | M | France | After 1^st^ lockdown | 2016 | Staff consultant | Outpatient (Public and private practice) |
| 16 | 44 | F | Finland | Lockdown ended  Social distanciang | 2020 | head of department | Outpatient |
| 17 | 37 | M | France | After 1^st^ lockdown | 2013 | Senior physician | Liaison-consultation  Outpatient |
| 18 | 54 | F | Switzerland | Progressive ending of the lockdown | 1992 | Head of department | Outpatient  psychotherapy |
| 19 | 38 | M | Indonesia | Social restriction | 2015 | Senior fellow | Outpatient (Public and Private practice)  Lecture |
| 20 | 42 | M | Greece | Open (except hotel) | 2013 | consultant | Outpatient (NGO and private practice)  psychotherapy |
| 21 | 45 | F | France | After 1^st^ lockdown | 2004 | Senior physician | Outpatient, day-hospital |
| 22 | 58 | F | Malaysia | Rehabilitation  phase | 2004 | Consultant | Outpatient  Psychotherapy  supervision |
| 23 | 35 | F | Belgium | Lockdown ended | 2015 | Chief of resident | Outpatient  Day hospital  psychotherapy |
| 24 | 47 | F | Mexico | Lockdown | 1997 | Senior fellow | Liaison-consultation  Outpatient (public and private practice)  research |
| 25 | 35 | F | Mexico | lockdown | 2016 | Senior fellow | Outpatient |
| 26 | 30 | M | Ukraine | Lockdown for children | 2017 | consultant | Outpatient  research |
| 27 | 46 | F | Nigeria | Progressive ending of the lockdown Curfew  Social distancing | 2016 | Consultant/ head of unit | outpatient  Lecturer  research |
| 28 | 70 | M | USA | lockdown | 1978 | Professor | Outpatient  Research |
| 29 | 45 | M | japan | Not official but effort for social distance | 2004 | Medical Director | outpatient |
| 30 | 66 | M | Cambogia | Lockdown ended | 1994 | Medical director | outpatient |
| 31 | 48 | F | Hungry | Lockdown Ended  masks in transport and shopping, Social distancing | 2004 | Associate professor/head of department | Inpatient and outpatient |
| 32 | 54 | F | New Zealand | Level one  (22 cases)  No social distancing | 2006 | Consultant | Outpatient  Community clinic  Youth forensic  Private practice |
| 33 | 47 | H | Congo-Kinshasa | Social restrictions  Schools and restaurants closed | 2010 | Consultant | Outpatient (ER, public and Private practice) |
| 34 | 40 | F | USA | lockdown | 2017 | Trainee director | outpatient |
| 35 | 49 | M | Japan | No Lockdown | 1997 | Medical director | Outpatient and inpatient |
| 36 | 33 | F | Germany | No lockdown  Masks  Social distancing | 2019 | Senior physician | outpatient  Day hospital |
| 37 | 64 | M | United-Kingdom | Lockdown ended  Masks in closed spaces  Social distancing  Local lockdown | 1990 | Senior Lecturer | Outpatient  Research  Lecture |
| 38 | 52 | M | United-Kingdom | Lockdown ended  Masks in closed spaces  Social distancing  Local lockdown | 1997 | Senior physician | Outpatient  Research  Lecture |
| 39 | 61 | F | Portugal | Lockdown ended  Masks in closed spaces and hospital  Social distancing | 1985 | Senior physician | Inpatient  Liaison-Consultation  Outpatient  psychotherapy |
